# Acute Adrenal Insufficiency Caused by Mental Stress in a Patient With Adrenocorticotropic Hormone Deficiency

**DOI:** 10.7759/cureus.36933

**Published:** 2023-03-30

**Authors:** Hidefumi Sueoka, Kento Ikegawa, Yukihiro Hasegawa

**Affiliations:** 1 Division of Endocrinology and Metabolism, Tokyo Metropolitan Children’s Medical Center, Tokyo, JPN; 2 Clinical Research Support Center, Tokyo Metropolitan Children’s Medical Center, Tokyo, JPN

**Keywords:** stress dose of steroids, acth deficiency, adrenal crisis, glucocorticoid, hydrocortisone, emotional stress, mental stress, adrenal insufficiency

## Abstract

In patients with chronic adrenal insufficiency, physical stress increases the requirement for glucocorticoid therapy. Although mental stress may cause acute adrenal insufficiency, it is debatable how patients should be treated when experiencing mental stress. Here, we report the case of a female patient with septo-optic dysplasia who had been treated for adrenocorticotropic hormone deficiency since infancy. After her grandfather died when she was 17 years old, she complained of nausea and stomach pain. Her symptoms failed to improve despite treatment with stress doses of oral hydrocortisone and self-administered glucagon injection. Her general condition improved after she began receiving continuous hydrocortisone and glucose infusions. Glucocorticoid stress doses should be given early if a patient is likely to experience mental stress.

## Introduction

Patients with chronic adrenal insufficiency are unable to produce sufficient cortisol and are at risk of acute adrenal insufficiency when under physical stress. Therefore, increasing the glucocorticoid dose during physical stress, such as that caused by febrile illness or gastroenteritis, is critical [1,2]. On the other hand, some guidelines disrecommend increasing the glucocorticoid dose for mental stress [1,2]. Here, we present the case of a female patient with septo-optic dysplasia in whom acute adrenal insufficiency developed after she experienced mental stress caused by her grandfather’s death. The term *mental stress* used in this study is synonymous with *psychic distress*, *psychological stress*, and *emotional stress*, which have been used in previous reports [3-5]. These previous studies showed that mental stress might cause acute adrenal insufficiency in patients with chronic adrenal insufficiency, and, therefore, stress dosing may be necessary for mental stress [3-5].

## Case presentation

The patient, a 17-year-old female, had received a diagnosis of septo-optic dysplasia in infancy and was being treated for combined hypopituitarism. She was receiving oral hydrocortisone therapy for adrenocorticotropic hormone (ACTH) deficiency and was also being treated for growth hormone (GH), thyroid-stimulating hormone, and vasopressin deficiencies. Her height and weight were 147.0 cm (−2.1 standard deviation [SD]) and 50.2 kg (−0.4 SD) at the age of 17.

She experienced frequent episodes of acute adrenal insufficiency during mild illnesses and had poor oral food intake in infancy and early childhood, which led to her being hospitalized more than 20 times by the age of five years. When ill, she was instructed to self-administer a stress dose of hydrocortisone 100 mg/m^2^/day orally. Furthermore, her parents were trained to administer intramuscular hydrocortisone injections. Self-injection of glucagon was also prescribed to prevent hypoglycemia. As she grew older, the number of hospitalizations decreased.

After the patient’s father died during her infancy, her paternal grandfather cared for her in loco parentis, providing emotional support in times of need. Two days before her grandfather’s death, she experienced a mild headache and abdominal pain, which improved with oral analgesics. On the day of his death, she self-administered one stress dose of hydrocortisone 25 mg. Two days after his death, her nausea and abdominal pain deteriorated, and hypoglycemia (blood glucose level: 64 mg/dL) developed, prompting her to visit a local clinic after she had given herself an intramuscular injection of hydrocortisone 100 mg and glucagon 1 mg. Her symptoms failed to improve, and she was transferred to our hospital after she additionally received an injection of hydrocortisone 50 mg. Despite appearing fatigued, she had a Glasgow Coma Scale score of 15 on admission, indicating no impairment of consciousness. Table 1 shows the laboratory data. Although the patient had already received glucose and saline intravenously at the previous clinic, her blood glucose and serum sodium values were relatively low. Her symptoms gradually improved after she began receiving a continuous, intravenous infusion of 7.5% glucose solution and hydrocortisone 100 mg/day. She was able to resume regular meals after half a day and was discharged the next day.

**Table 1:** Laboratory data on arrival.

Laboratory data	Result	Reference range
Sodium (mEq/L)	134	138–144
Potassium (mEq/L)	4.2	3.7–4.7
Blood glucose (mg/dL)	85	70–109
Blood urea nitrogen (mg/dL)	11.6	6.8–11.6
Creatinine (mg/dL)	0.51	0.35–0.75
C-reactive protein (mg/dL)	0.01	<0.30

## Discussion

Likely, the mental stress caused by the death of the patient’s grandfather triggered acute adrenal insufficiency in our patient even in the absence of any apparent physical stress.

As in the present case, acute adrenal insufficiency is diagnosed based on signs and symptoms, such as hypotension, acute abdominal symptoms, nausea or vomiting, and laboratory abnormalities, including hyponatremia, hyperkalemia, and hypoglycemia, although the diagnosis is sometimes challenging [4]. Our patient had experienced recurrent episodes of acute adrenal insufficiency stemming from physical stress. The initial symptoms in these previous episodes were abdominal pain and nausea, which improved with continuous, intravenous hydrocortisone infusions. Furthermore, her C-reactive protein was not elevated, refuting the possibility of infection. While the patient had other, concurrent anterior pituitary hormone deficiencies, including GH deficiency, which may have contributed to the prolonged hypoglycemia and malaise, she was admitted for adrenal insufficiency stemming from the mental stress induced by her grandfather’s death. The present episode is noteworthy in that it was triggered by mental, rather than physical, stress, unlike the previous episodes.

Current guidelines in the United States and Japan disrecommend increasing glucocorticoid administration during mental stress in patients with chronic adrenal insufficiency [1,2]. On the other hand, some studies have suggested that mental stress can trigger an adrenal crisis [3], and some experts recommend increasing the hydrocortisone dose slightly if a patient is experiencing mental stress [5]. The European Reference Network on rare endocrine conditions recommends increasing the hydrocortisone dose in times of mental stress caused by events such as a dental visit, job interview, or death in the family [6].

## Conclusions

We presented a case of acute adrenal insufficiency, which occurred after the patient experienced the death of a close relative. Mental stress can trigger acute adrenal insufficiency in patients with chronic adrenal insufficiency. Early administration of an increased glucocorticoid stress dose should be considered if a patient is likely to experience mental stress even in the absence of any apparent physical stress.
